# Supplementary figures and images for: Prevalence of biofilms in Candida spp. bloodstream infections: A meta-analysis
Source: PLoS One. 2022 Feb 3;17(2):e0263522. doi: 10.1371/journal.pone.0263522 (PMC8812928; doi:10.1371/journal.pone.0263522)

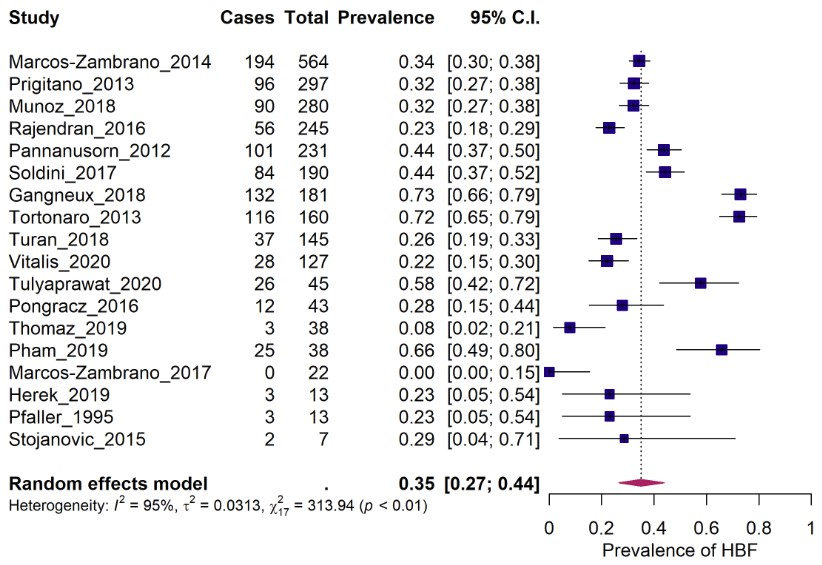

Supplement: S1 Fig — (TIF) [file pone.0263522.s001.tif]

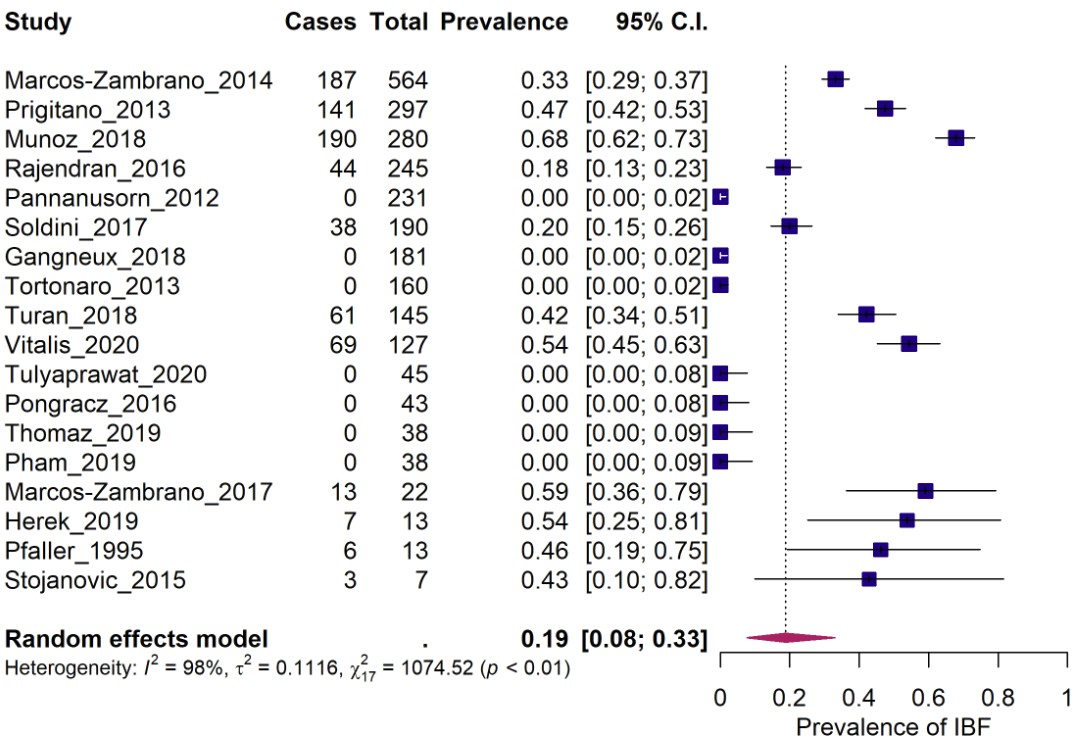

Supplement: S2 Fig — (TIF) [file pone.0263522.s002.tif]

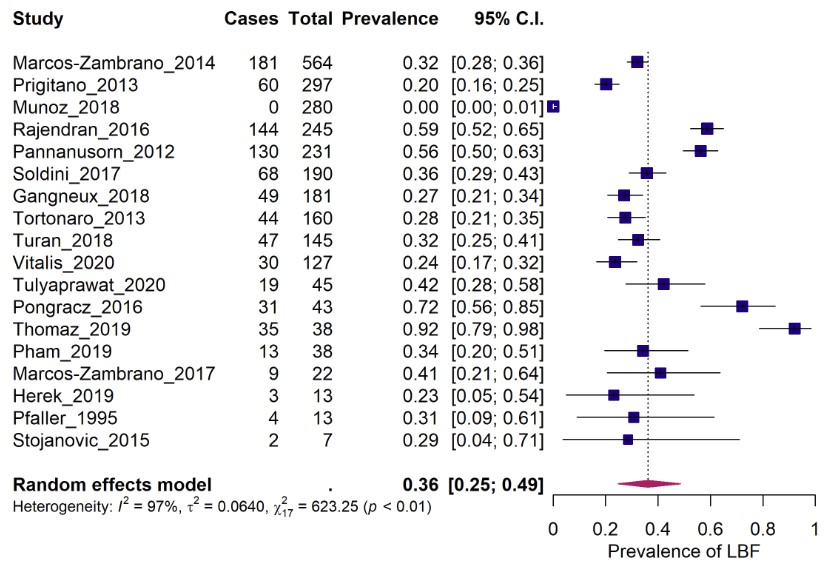

Supplement: S3 Fig — (TIF) [file pone.0263522.s003.tif]
