# Supplementary material for: Prevalence of biofilms in Candida spp. bloodstream infections: A meta-analysis
Source: PLoS One. 2022 Feb 3;17(2):e0263522. doi: 10.1371/journal.pone.0263522 (PMC8812928; doi:10.1371/journal.pone.0263522)
Supplement: S1 Table — (DOCX) [file pone.0263522.s004.docx]

S1 Table. Subgroup analysis between different *Candida* species and biofilm-forming capability.

| Species | k | N | BF strains  (n) | Biofilm rate, % (95% CI) | k | Prevalence, % (95% CI) | | | | | |
| --- | --- | --- | --- | --- | --- | --- | --- | --- | --- | --- | --- |
|  |  |  |  |  |  | **n** | **HBF** | **n** | **IBF** | **n** | **LBF** |
| *C. albicans* | 22 | 2105 | 1461 | 71.2 (52.4 – 87.1) | 12 | 496 | 33.0 (22.9-43.9) | 399 | 20.7(6.3-39.9) | 396 | 34.3 (17.6-53.2) |
| No *C. albicans* | 26 | 2140 | 1868 | 88.1(81.8-93.5) | 16 | 566 | 36.0(02.6-47.0) | 356 | 17.1(8.4-27.5) | 607 | 31.4(20.7-43.1) |
| *C. glabrata* | 17 | 463 | 387 | 76.2 (55.7-92.6) | 8 | 104 | 20.9 (0.2 – 57.7) | 70 | 21.1(0.3-55.7) | 180 | 41.1(6.9-80.6) |
| *C. tropicalis* | 17 | 361 | 331 | 96.2 (89.1-99.4) | 9 | 135 | 68.8 (59.4 – 76.9) | 34 | 13.0(2.7-27.8) | 31 | 9.58(1.1-22.8) |
| *C. parapsilosis* | 20 | 853 | 744 | 87.5 (74.3-96.9) | 12 | 199 | 34.6 (22.8 – 47.4) | 200 | 22.2(8.7-39.0) | 233 | 33.5(18.6-49.9) |
| *C. krusei* | 10 | 86 | 68 | 85.7 (62.9 – 99.6) | 6 | 8 | 18.9 (0.0 – 58.4) | 15 | 21.6 (0.0-70.7) | 20 | 43.1(4.2-87.2) |
| *Other species | 20 | 377 | 338 | 89.9 (74.7-99.4) | 14 | 121 | 29.6 (11.6 – 50.5) | 37 | 12.2(0.0-36.0) | 143 | 40.2(23.1-58.3) |
| p-value** |  |  |  |  |  | < 0.0001 | | 0.9679 | | 0.0429 | |

*Other species includes *C. dublinensis* (n=14), *C. quilliermondi* (n=25), *C. lusitaniae* (n=10), *C. haemulonii (*n=4), *C. keyfr* (n=4), *C. lypolitica* (n=1), *C. pelliculosa* (n=1) and unreported species (n=318)

** Test for subgroup differences
