# Supplementary material for: Prevalence of biofilms in Candida spp. bloodstream infections: A meta-analysis
Source: PLoS One. 2022 Feb 3;17(2):e0263522. doi: 10.1371/journal.pone.0263522 (PMC8812928; doi:10.1371/journal.pone.0263522)
Supplement: S1 File — (DOCX) [file pone.0263522.s005.docx]

| **Section and Topic** | **Item #** | **Checklist item** | **Location where item is reported** |
| --- | --- | --- | --- |
| **TITLE** | | |  |
| Title | 1 | Identify the report as a systematic review. | Line 1 |
| **ABSTRACT** | | |  |
| Abstract | 2 | See the PRISMA 2020 for Abstracts checklist. | Lines 18 - 52 |
| **INTRODUCTION** | | |  |
| Rationale | 3 | Describe the rationale for the review in the context of existing knowledge. | Lines 89 - 98 |
| Objectives | 4 | Provide an explicit statement of the objective(s) or question(s) the review addresses. | Lines 22-23; 98 – 100, and  Suplementary 2 (Sheet entitled **PICO-Question**, in the Metanalysis Process Excel file) |
| **METHODS** | | |  |
| Eligibility criteria | 5 | Specify the inclusion and exclusion criteria for the review and how studies were grouped for the syntheses. | Lines 490– 494, and  Suplementary 2 (Sheets entitled **Inclusion Criteria** and **Key Question,** in the Metanalysis Process Excel file) |
| Information sources | 6 | Specify all databases, registers, websites, organisations, reference lists and other sources searched or consulted to identify studies. Specify the date when each source was last searched or consulted. | Line 491 – 453, and  Supplementary 2 (Sheet entitled **Search Strategy**, in the Metanalysis Process Excel file) |
| Search strategy | 7 | Present the full search strategies for all databases, registers and websites, including any filters and limits used. | Supplementary 2 (Sheet entitled **Search Strategy,** in the Metanalysis Process Excel file) |
| Selection process | 8 | Specify the methods used to decide whether a study met the inclusion criteria of the review, including how many reviewers screened each record and each report retrieved, whether they worked independently, and if applicable, details of automation tools used in the process. | Llines 487 - 492, and  Supplementary 2 (Sheet entitled **Inclusion Criteria** and **Eligibility,** in the Metanalysis Process Excel file) |
| Data collection process | 9 | Specify the methods used to collect data from reports, including how many reviewers collected data from each report, whether they worked independently, any processes for obtaining or confirming data from study investigators, and if applicable, details of automation tools used in the process. | Llines 488 - 491, 498-499,and  Supplementary 2 (Sheet entitled **Data Extraction**, in the Metanalysis Process Excel file) |
| Data items | 10a | List and define all outcomes for which data were sought. Specify whether all results that were compatible with each outcome domain in each study were sought (e.g. for all measures, time points, analyses), and if not, the methods used to decide which results to collect. | Llines 491 - 497, and  Supplementary material Sheet entitled **Tabulation**, in the Metanalysis Process Excel file. |
|  | 10b | List and define all other variables for which data were sought (e.g. participant and intervention characteristics, funding sources). Describe any assumptions made about any missing or unclear information. | Table 2, and  Supplementary material Sheet entitled **Tabulation**, in the Metanalysis Process Excel file. |
| Study risk of bias assessment | 11 | Specify the methods used to assess risk of bias in the included studies, including details of the tool(s) used, how many reviewers assessed each study and whether they worked independently, and if applicable, details of automation tools used in the process. | Lines 530 – 534, and Figure 3 |
| Effect measures | 12 | Specify for each outcome the effect measure(s) (e.g. risk ratio, mean difference) used in the synthesis or presentation of results. | Figure 2 and description. |
| Synthesis methods | 13a | Describe the processes used to decide which studies were eligible for each synthesis (e.g. tabulating the study intervention characteristics and comparing against the planned groups for each synthesis (item #5)). | Lines 510 – 511, and  Supplementary material Sheet entitled **Tabulation** and **Key Question**, in the Metanalysis Process Excel file. |
|  | 13b | Describe any methods required to prepare the data for presentation or synthesis, such as handling of missing summary statistics, or data conversions. | Lines 463 - 464 |
|  | 13c | Describe any methods used to tabulate or visually display results of individual studies and syntheses. | Figure 1, Figure 2, Figure 3 and Table 1 |
|  | 13d | Describe any methods used to synthesize results and provide a rationale for the choice(s). If meta-analysis was performed, describe the model(s), method(s) to identify the presence and extent of statistical heterogeneity, and software package(s) used. | Lines 509 - 520 |
|  | 13e | Describe any methods used to explore possible causes of heterogeneity among study results (e.g. subgroup analysis, meta-regression). | Lines 532- 534 |
|  | 13f | Describe any sensitivity analyses conducted to assess robustness of the synthesized results. | Lines 532- 534 |
| Reporting bias assessment | 14 | Describe any methods used to assess risk of bias due to missing results in a synthesis (arising from reporting biases). | Lines 534  Figure 3 |
| Certainty assessment | 15 | Describe any methods used to assess certainty (or confidence) in the body of evidence for an outcome. | Lines 520 – 521,  Figure 2 |
| **RESULTS** | | |  |
| Study selection | 16a | Describe the results of the search and selection process, from the number of records identified in the search to the number of studies included in the review, ideally using a flow diagram. | Lines 102 – 109;  Figure 1. |
|  | 16b | Cite studies that might appear to meet the inclusion criteria, but which were excluded, and explain why they were excluded. | Supplementary 2 (Sheet entitled **Eligibility,** in the Metanalysis Process Excel file) |
| Study characteristics | 17 | Cite each included study and present its characteristics. | Supplementary 2 (Sheet entitled **Data Extraction**, in the Metanalysis Process Excel file) |
| Risk of bias in studies | 18 | Present assessments of risk of bias for each included study. | Figure 2,  Figure 3 |
| Results of individual studies | 19 | For all outcomes, present, for each study: (a) summary statistics for each group (where appropriate) and (b) an effect estimate and its precision (e.g. confidence/credible interval), ideally using structured tables or plots. | Lines 119 - 124  Table 1,  Figure 2, |
| Results of syntheses | 20a | For each synthesis, briefly summarise the characteristics and risk of bias among contributing studies. | Tables 3-6 |
|  | 20b | Present results of all statistical syntheses conducted. If meta-analysis was done, present for each the summary estimate and its precision (e.g., confidence/credible interval) and measures of statistical heterogeneity. If comparing groups, describe the direction of the effect. | Lines 35 – 49; 119 – 124,  Figure 2 |
|  | 20c | Present results of all investigations of possible causes of heterogeneity among study results. | Lines 417 - 422 |
|  | 20d | Present results of all sensitivity analyses conducted to assess the robustness of the synthesized results. | Lines 275 - 277 |
| Reporting biases | 21 | Present assessments of risk of bias due to missing results (arising from reporting biases) for each synthesis assessed. | Tables 3-6 |
| Certainty of evidence | 22 | Present assessments of certainty (or confidence) in the body of evidence for each outcome assessed. | Lines 121 -124, Lines 151 – 155, Lines 202 – 210,Lines 216 – 217, Lines 237 – 239,Lines 246 – 251, Lines 263 – 267,Lines 151 – 155, Lines 280 – 285. |
| **DISCUSSION** | | |  |
| Discussion | 23a | Provide a general interpretation of the results in the context of other evidence. | Lines 299 - 351 |
|  | 23b | Discuss any limitations of the evidence included in the review. | Lines 458 - 464 |
|  | 23c | Discuss any limitations of the review processes used. | N/A |
|  | 23d | Discuss implications of the results for practice, policy, and future research. | Lines 462 - 454 |
| **OTHER INFORMATION** | | |  |
| Registration and protocol | 24a | Provide registration information for the review, including register name and registration number, or state that the review was not registered. | N/A |
|  | 24b | Indicate where the review protocol can be accessed, or state that a protocol was not prepared. | Supplementary 2 |
|  | 24c | Describe and explain any amendments to information provided at registration or in the protocol. | N/A |
| Support | 25 | Describe sources of financial or non-financial support for the review, and the role of the funders or sponsors in the review. | In the online Funding Statement on PLOS ONE as: “This work was supported by COCIBA Research Grant 2018-2019 through project ID: 12260 entitled “Adhesión inicial y resistencia antimicrobiana de *Candida* sp. aisladas de la microbiota humana”, under regulations of the Ministry of Health of Ecuador (Contrato Marco de Acceso a los Recursos Genéticos No. MAE-DNB-CM-2016-0046). The funders had no role in study design, data collection and analysis, decision to publish, or preparation of the manuscript ” |
| Competing interests | 26 | Declare any competing interests of review authors. | In the online Conflict of Interest on PLOS ONE as: “The authors declare that the research was conducted in the absence of any commercial or financial relationships that could be construed as a potential conflict of interest.”. |
| Availability of data, code and other materials | 27 | Report which of the following are publicly available and where they can be found: template data collection forms; data extracted from included studies; data used for all analyses; analytic code; any other materials used in the review. | Supplementary 2, Supplementary 3, Supplementary 4 |

*From:*  Page MJ, McKenzie JE, Bossuyt PM, Boutron I, Hoffmann TC, Mulrow CD, et al. The PRISMA 2020 statement: an updated guideline for reporting systematic reviews. BMJ 2021;372:n71. doi: 10.1136/bmj.n71

For more information, visit: <http://www.prisma-statement.org/>
