# Supplementary material for: Prevalence of biofilms in Candida spp. bloodstream infections: A meta-analysis
Source: PLoS One. 2022 Feb 3;17(2):e0263522. doi: 10.1371/journal.pone.0263522 (PMC8812928; doi:10.1371/journal.pone.0263522)
Supplement: Attachment — Submitted filename: Response to Reviewers-Final.docx [file pone.0263522.s009.docx]

**Revised Manuscript**

**Answer to the Reviewer’s comments**

Based on the following comments and suggestions of **Reviewer 2**, we have made new modifications to the previous version of the manuscript. These additional changes are also shown in the newly revised manuscript with track changes.

# **Comments to the Author**

1. If the authors have adequately addressed your comments raised in a previous round of review and you feel that this manuscript is now acceptable for publication, you may indicate that here to bypass the “Comments to the Author” section, enter your conflict of interest statement in the “Confidential to Editor” section, and submit your "Accept" recommendation.

Reviewer #2: (No Response)

2. Is the manuscript technically sound, and do the data support the conclusions?

The manuscript must describe a technically sound piece of scientific research with data that supports the conclusions. Experiments must have been conducted rigorously, with appropriate controls, replication, and sample sizes. The conclusions must be drawn appropriately based on the data presented.

Reviewer #2: Yes

3. Has the statistical analysis been performed appropriately and rigorously?

Reviewer #2: Yes

4. Have the authors made all data underlying the findings in their manuscript fully available?

The [PLOS Data policy](https://urldefense.proofpoint.com/v2/url?u=http-3A__www.plosone.org_static_policies.action-23sharing&d=DwMGaQ&c=21GvzBjUVuezJWxqZ0YzE5zwoFbI4Gsp4f3hKxgRYPQ&r=iurDEgzFS80IoSdzfLfy_Vp7NYKtvAsn-saMgK4v1xQ&m=XXsW6WHYRx7zGSDyT7kFodLY5vqVZvh8r_XD7NHvgD8&s=U9sSpD3f7UqScINHy3AJChVuURJ0rBKR-RYg82xtA7A&e=) requires authors to make all data underlying the findings described in their manuscript fully available without restriction, with rare exception (please refer to the Data Availability Statement in the manuscript PDF file). The data should be provided as part of the manuscript or its supporting information, or deposited to a public repository. For example, in addition to summary statistics, the data points behind means, medians and variance measures should be available. If there are restrictions on publicly sharing data—e.g. participant privacy or use of data from a third party—those must be specified.

Reviewer #2: Yes

5. Is the manuscript presented in an intelligible fashion and written in standard English?

PLOS ONE does not copyedit accepted manuscripts, so the language in submitted articles must be clear, correct, and unambiguous. Any typographical or grammatical errors should be corrected at revision, so please note any specific errors here.

Reviewer #2: Yes

**Reviewer 2 Report**

**Author’s answer** –We thank the Reviewer 2 for their insight and supportive comments. These amendments are detailed in the following answer to Reviewer 2 and illustrated in the revised manuscript with track changes. We hope that Reviewer 2 finds this version suitable for publication in *PLOS ONE* journal.

# **Reviewer #2:**

6. Review Comments to the Author

**Reviewer #2:** The authors did a very good job addressing the issues from the first review. The only remaining suggestion I have is to properly cite the R packages in the methods section, and add the following references to the bibliography:

For "meta"

Balduzzi S, Rücker G, Schwarzer G (2019). “How to perform a meta-analysis with R: a practical tutorial.” Evidence-Based Mental Health, 153–160.

For "metafor"

Viechtbauer W (2010). “Conducting meta-analyses in R with the metafor package.” Journal of Statistical Software, 36(3), 1–48. https://doi.org/10.18637/jss.v036.i03.

For "poibin"

Hong, Y. (2013). On computing the distribution function for the Poisson binomial distribution. Computational Statistics & Data Analysis, Vol. 59, pp. 41-51.

For "stringr"

Wickham H (2021). stringr: Simple, Consistent Wrappers for Common String Operations. http://stringr.tidyverse.org, https://github.com/tidyverse/stringr.

**Author’s answer**– As well-appointed by Reviewer 2, we properly cited the R packages in the methods section and added the references to the bibliography (Please check these amendments on page 30 lines 518-519 and page 41 lines 745-753 of the revised manuscript with track changes, respectively). Also, we want to thank you Reviewer 2 to give us the proper citations of the R packages that allowed us to quickly rectify our mistake.
